# Influence of reconstruction settings on the performance of adaptive thresholding algorithms for FDG‐PET image segmentation in radiotherapy planning

**DOI:** 10.1120/jacmp.v12i2.3363

**Published:** 2011-01-30

**Authors:** Roberta Matheoud, Patrizia Della Monica, Gianfranco Loi, Luca Vigna, Marco Krengli, Eugenio Inglese, Marco Brambilla

**Affiliations:** ^1^ Department of Medical Physics University Hospital “Maggiore della Carità” Novara Italy; ^2^ Department of Radiotherapy University Hospital “Maggiore della Carità” Novara Italy; ^3^ Department of Nuclear Medicine University Hospital “Maggiore della Carità” Novara Italy

**Keywords:** FDG‐PET/CT, radiation treatment planning, functional imaging, target volume definition

## Abstract

The purpose of this study was to analyze the behavior of a contouring algorithm for PET images based on adaptive thresholding depending on lesions size and target‐to‐background (TB) ratio under different conditions of image reconstruction parameters. Based on this analysis, the image reconstruction scheme able to maximize the goodness of fit of the thresholding algorithm has been selected. A phantom study employing spherical targets was designed to determine slice‐specific threshold (TS) levels which produce accurate cross‐sectional areas. A wide range of TB ratio was investigated. Multiple regression methods were used to fit the data and to construct algorithms depending both on target cross‐sectional area and TB ratio, using various reconstruction schemes employing a wide range of iteration number and amount of postfiltering Gaussian smoothing. Analysis of covariance was used to test the influence of iteration number and smoothing on threshold determination.

The degree of convergence of ordered‐subset expectation maximization (OSEM) algorithms does not influence TS determination. Among these approaches, the OSEM at two iterations and eight subsets with a 6–8 mm post‐reconstruction Gaussian three‐dimensional filter provided the best fit with a coefficient of determination $R^{2}=0.90$ for cross‐sectional areas ≤ 133 mm ^2^ and $R^{2}=0.95$ for cross‐sectional areas > 133 mm ^2^. The amount of post‐reconstruction smoothing has been directly incorporated in the adaptive thresholding algorithms. The feasibility of the method was tested in two patients with lymph node FDG accumulation and in five patients using the bladder to mimic an anatomical structure of large size and uniform uptake, with satisfactory results.

Slice‐specific adaptive thresholding algorithms look promising as a reproducible method for delineating PET target volumes with good accuracy.

PACS numbers: 87.57.nm, 87.55.D‐, 87.57.uk

## I. INTRODUCTION

^18^F‐fluorodeoxyglucose (FDG) positron emission tomography (PET) was incorporated into target volume delineation by many groups, and various methods for target volume definition are currently in use.^(1)^ Methods for segmentation based on contrast‐oriented contouring algorithms^(^ ^2^ ^–^ ^4^ ^)^ have been developed independently by many groups and validated in patient data both in head and neck^(5)^ and in lung cancer^(6)^ with satisfactory results. These methods rely on a system‐specific calibration curve, which depends on target‐to‐background (TB) ratio.^(^ ^2^ ^–^ ^3^ ^,^ ^7^ ^)^ When the full range of clinically relevant volumes – including also small volumes – is considered, the calibration curve also results dependent on target size.^(^ ^4^ ^,^ ^8^ ^–^ ^10^ ^)^ It was demonstrated that among acquisition parameters, emission scan duration and background activity concentration, both related to total number of counts and to the level of image noise, did not result as significant predictors in threshold (TS) determination.^(^ ^6^ ^,^ ^10^ ^)^ Recently it was also demonstrated^(11)^ that adaptive thresholding algorithms are not influenced by the different conditions of attenuation and scatter. Thus, the calibration curve need not to be specifically devised for each anatomical site representing different conditions of attenuation and scatter, and may be applied irrespective of the phantom used in its derivation. The effect of PET image reconstruction parameter has not been fully explored in literature. The use of iterative image reconstruction algorithms have demonstrated marked improvement in image quality.^(12)^ Ordered‐subset expectation maximization (OSEM) algorithm,^(13)^ which is related to but much faster than maximum‐likelihood expectation maximization, became the dominant iterative reconstruction procedure in emission tomography.^(14)^ The role of OSEM EM‐equivalent iteration number^(15)^ has not yet been investigated while, as indicated by other authors, a wider post‐reconstruction smoothing filter results in a shifted curve such that the same measured volume is obtained for a higher percent threshold contour level.^(^ ^2^ ^,^ ^8^ ^)^

However, until now the amount of smoothing has not been directly included in a segmentation algorithm. By first principles, iteration schemes using a large number of iterations should increase the convergence of the algorithm, usually at the expense of increased noise. This is relevant, especially in smaller regions since this can clearly affect the maximum value in the target and, therefore, the percentage threshold. For instance, Jaskowiak et al.^(16)^ demonstrated a significant difference both in average and maximum standardized uptake value across different iteration groups, while Fin et al.^(17)^ demonstrated progressively lower contrast‐to‐noise ratio and higher contrast recovery as the number of iterations increases.

Our aim is to study the behavior of a contouring algorithm based on adaptive thresholding depending on target size and TB ratio under different conditions of image reconstruction parameters. Based on this analysis, the image reconstruction scheme which maximizes the goodness of fit and the robustness of the thresholding algorithm in a phantom experiment was selected, and the reconstruction settings which have a relevant role in threshold prediction were directly incorporated in the algorithms. Finally, the feasibility of the method was tested in two patients with lymph node FDG accumulation, and in five consecutive patients using the bladder to simulate an anatomical structure of large size and uniform uptake.

## II. MATERIALS AND METHODS

### A. Phantoms

Measurements were performed on two phantom sets (Data Spectrum Corporation, Hillsborough, NC). The first is a modification of the IEC Body Phantom Set. The IEC phantom alone tends to overestimate true and random count rates and to underestimate scatter fraction (SF) common to clinical patient scanning. Therefore additional attenuation and scatter material (an annular ring of 3 cm water bags) were added to better approximate typical clinical count rates.^(18)^ In this phantom six fillable polymethilmetacrilate spheres with internal diameters of 10, 13, 17, 22, 28 and 37 mm and wall thickness of 1 mm are inserted. A supplemental set of four micro‐hollow spheres of 4.1, 4.7, 6.5, 8.1 mm internal diameters was positioned at the bottom of the phantom. To simulate the presence of activity external to the field of view, a NEMA Scatter Phantom Set was positioned at the end of the modified IEC phantom. The inner plastic capillary was filled in order to have an equivalent activity concentration in the whole scatter phantom as in the one of the main chamber of the modified IEC phantom, as requested by the NEMA‐01 standard and to approximate an average condition that can be encountered clinically.^(19)^

The background of the modified IEC phantom was filled with 5.4 kBq/ml activity concentration of ${}^{18}F‐\text{FDG}$. The source‐to‐background ratios, as determined by the dose calibrator, were set to 2.5, 4.2, 6.6, 8.1, 16.6, 24.7, 35, 47, 55 and 70 in different acquisition sessions. Overall, 10 statistically independent fully three‐dimensional coincidence sinograms were acquired. The partial‐volume and spillover effects influence the measured source activity concentration in the sphere. The measured TB ratio obtained from PET images differed from prepared source‐to‐background ratio as determined by the dose calibrator. TB ratios were determined in the reconstructed image as the maximum pixel intensity in a region‐of‐interest (ROI) encircling the cross‐sectional area of the target, divided by the average pixel intensity of ROIs surrounding the sphere. These TB ratios ranged from 70 down to 1.5 and were within the full range observed in patients. ROIs analysis was performed, as previously described,^(20)^ by means of an automatic routine developed using IDL 6.1 (Research System, Inc.) to avoid the influence of the operator in ROIs dimensioning and to minimize the influence of the operator in the ROIs positioning. Briefly, a pattern of six ROIs of fixed dimensions (diameters equal to the physical ID of the spheres) and fixed relative distances is presented to the operator who can only rotate and translate the pattern to establish its correct position over the hot spheres in the slice. The ROIs analysis tool permits movement of the ROIs pattern in increments of less than 1 mm. The operator is also requested to position a pattern of twelve 37 mm background ROIs at a distance of 15 mm from the edge of the phantom but no closer than 15 mm to any sphere. The positioning and dimensioning of the smaller ROIs (10, 13, 17, 22, and 28 mm) on background were done automatically from the placement of the original 12 background ROIs. The same pattern of 12 background ROIs was automatically positioned at a distance of ± 1 and ± 2cm from the central slice for a total of 60 background ROIs, as prescribed by NEMA recommendations. The same analysis is repeated for the four micro‐hollow spheres by choosing a different central slice.

### B. Phantom acquisition and PET image reconstruction

**Figure 1:**
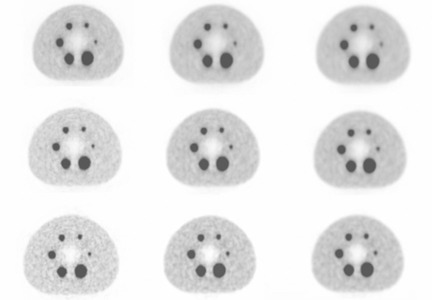
Changes in image appearance of different targets as number of iterations is increased from 16 (top row) to 64 (bottom row) and as smoothing kernel is increased from FWHM=4 mm (left column) to FWHM=8 mm (right column).

Images were acquired with the Biograph 16 Hi‐Rez PET/CT scanner (Siemens Medical Solutions). A 16.2 cm axial field of view is covered by 81 image planes with slice thickness of 2 mm for each bed. The scanner transverse spatial resolution and axial resolution are 4.6 and 5.1 mm FWHM at 1 cm radial position. Both axial and transaxial FWHM values degraded by about 0.8 mm when moving form 1 to 10 cm away from the central axis of the scanner.^(20)^ The emission scan duration was set to 5 min/bed according to clinical acquisition protocols used in our institution for radiotherapy planning. The IEC phantom has a flat surface on the side that must be positioned on the examination table, so no rotations of the IEC phantom can occur. The same positioning of the phantom was ensured through laser localizer and a scout CT acquisition. PET image reconstruction was performed after Fourier rebinning (FORE) with attenuation weighted OSEM‐iterative reconstruction with nine possible combinations of EM‐equivalent iteration number and amount of post‐reconstruction Gaussian three‐dimensional filter, as shown in Table 1. The resulting PET image had a matrix size of 256×256 pixels (voxel size 2.6×2.6×2 mm^3^). Figure 1 shows images of different targets with internal diameters ranging from 10 to 37 mm and acquired with a source‐to‐background ratio of 8.1. This example shows continuing changes in image appearance from 16 iterations to 64 iterations, for each level of smoothing. The data show progressively noisier images but with less smoothing and more spatial features as the number of iterations increases.

**Table 1:** Parameters of OSEM iterative reconstructions.

	*EM‐equivalent Iteration Number*	*Gaussian Smoothing FWHM (mm)*
2i8s4mm	16	4
2i8s6mm	16	6
2i8s8mm	16	8
4i8s6mm	32	4
4i8s6mm	32	6
4i8s8mm	32	8
8i8s4mm	64	4
8i8s6mm	64	6
8i8s8mm	64	8

### C. Data analysis

TS were determined as a percentage of the maximum intensity in the cross section area of the spheres. Target cross sections of area A were selected precisely in the middle of the spheres, which represents the largest cross section of the sphere, by using the inherently co‐registered CT scan. TS were also determined on the edges of the larger spheres to build a “validation” sample in order to assess the reliability of the regression models. The values of TS were entirely based on the apparent activity concentration in the images and not on the known activities actually placed in the spheres. To find the TS value that yielded the area A best matching the true value, the cross section was auto‐contoured in the attenuation corrected slices varying TS in steps of 1%, until the area so determined differed by less than 10 mm^2^ versus its known physical value. The threshold versus cross‐sectional area and TB plot was split and fitted into different functional forms, as already performed in previous studies,^(^ ^9^ ^–^ ^10^ ^)^ for each reconstruction strategy. One hundred and thirty‐three mm^2^ was selected as a separator of the data due to the resolution characteristics of our scanner.

### D. Statistical analysis

The relationship between the best threshold of intensity $\left(Y_{\text{ij}}\right)$ that provides the most accurate cross‐sectional area of the spheres and the variables $X_{1\text{ij}}$ (defined as 1−1/TB) and $X_{2\text{ij}}$ (defined as target cross section A), both linearly related to $Y_{\text{ij}}$ ^(10)^ were established using multiple linear regression methods for each combination of EM‐equivalent iteration number (i) and amount of post‐reconstruction Gaussian filtering (j), using the model:

(1) $$Y_{\text{ij}}=B_{0}+B_{1}\times sphere\;A(mm^{2})+B_{2}\times (1-\frac{1}{TB})+E$$

where $B_{0},B_{1}$ and $B_{2}$ are the regression coefficients that need to be estimated and *E* is the error term. The weight of different X‐variables in explaining Y was quantified by means of standard regression coefficients^(21)^ $\beta_{i}=B_{i}\times \sqrt{\sum x_{i}^{2}/\sum y^{2}}$ which can be used as a measure of relative importance, with the Xs ranked in order of the sizes of these coefficients. Goodness of fit for each reconstruction strategy was expressed using the adjusted coefficient of determination $\left(R^{2}\right)$, which is the proportion of variability in a data set that is accounted for by the statistical model, and provides a measure of how well future outcomes are likely to be predicted by the model. The reliability of the regression models was assessed by using the shrinkage on cross validation coefficients^(21)^ $R^{2}-R^{2}{}_{*}$ where $R^{2}{}_{*}$ is obtained through univariate correlation of the TS values measured on the validation sample and the predicted TS values obtained using the regression models.

Analysis of covariance methods^(21)^ were employed to compare the impact of different image reconstruction schemes on TS. Analysis of covariance model allows the simultaneous assessment of factors over the dependent variable.

Finally, multiple regressions models were built in order to directly incorporate the reconstruction parameters, which proved to be relevant in threshold prediction.

It must be emphasized that the models obtained should not be extrapolated outside the range of predictors values in which they have been determined.

Statistical analysis was performed using the software STATISTICA 6.0 (StatSoft Inc, Tulsa, OK).

### E. Patients

The feasibility of the method was tested in two patients with lymph node FDG accumulation and in five consecutive patients using the bladder to mimic an anatomical structure of large size and uniform uptake.

After injection of 4 MBq of ${}^{18}F‐\text{FDG}$ per kg of body weight, patients were rested for a period of about 60 minutes. Emission images ranging from the proximal femur to the base of the skull were acquired for 5 minutes per bed position. Field of view was of 50 cm with a matrix of 512×512 pixels for CT and of 256×256 for PET. PET image reconstruction was performed after FORE‐OSEM iterative reconstruction with two iterations, eight subsets and 8 mm Gaussian post‐reconstruction smoothing. The gross target volumes were delineated firstly on CT $\left({\text{GTV}}_{\text{CT}}\right)$ and then on PET $\left({\text{GTV}}_{\text{PET}}\right)$ images using the algorithms based on adaptive thresholding.

## III. RESULTS

**Figure 2:**
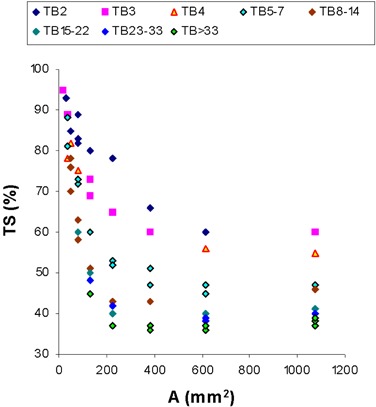
TS that produce correct target delineation over the full range of the sphere cross‐sectional areas examined. The dependence upon TB ratio is also represented in this graph. Reconstruction algorithm: eight subsets and two iterations with a Gaussian smoothing filter with FWHM=8 mm.

Figure 2 shows a representative example of TS required to produce correct target delineation over the full range of the sphere cross‐sectional areas examined. The dependence upon TB ratio is also represented in this graph. PET reconstructions were accomplished with iterative algorithm of OSEM obtained with eight subsets and two iterations with a Gaussian smoothing filter with a width of 8 mm. Different reconstruction strategies exhibit similar trends (not shown).

### A. Algorithm for cross section A $\leq \;133\;{\text{mm}}^{2}$

The regression equation that best summarizes the results obtained in a multiple regression model with TS as predicted variable and TB ratio and sphere A as predictor variables may be written as:

(2) $$TS(\%)=B_{0}+B_{1}\times sphereA\;\left(mm^{2}\right)+B_{2}\times (1-\frac{1}{TB})$$

The adjusted coefficient of determination $R^{2}$, together with the estimated and standard regression coefficients, were reported in Table 2 for each reconstruction strategy. Both sphere A and (1−1/TB) resulted as statistically significant predictors of TS for each reconstruction algorithm examined. TS diminishes with increasing sphere A and with increasing TB ratios. With the exception of 2i8s4mm algorithm, the functional dependence of TS on TB assessed by standard regression coefficients $\beta_{\left(1-1/\text{TB}\right)}$ is lower than the influence of cross‐sectional area, assessed by $\beta_{A}$ for the small volumes corresponding to sphere A ≤ 133 mm ^2^. As for the coefficient of determination $R^{2}$, only a slight diminishing trend can be observed with increasing EM‐equivalent iteration number, while no clear tendency emerges as for the amount of Gaussian smoothing.

**Table 2:** Results of multiple linear regression analysis with models fitted for sphere A $\leq \;133\;{\text{mm}}^{2}$.

*Reconstruction Strategy*	*R2*		β	*Standard Error of* β	*B*	*Standard Error of* β
2i8s4mm	0.91	Intercept			138.0	4.92
		Sphere A	−0.61	0.055	−0.23	0.02
		1−1/TB	−0.71		−74.7	5.72
2i8s6mm	0.91	Intercept			129.8	4.16
		Sphere A	−0.62	0.055	−0.25	0.02
		1−1/TB	−0.57		−56.7	5.43
2i8s8mm	0.90	Intercept			127.9	3.82
		Sphere A	−0.64	0.060	−0.23	0.02
		1−1/TB	−0.60		−48.7	4.88
4i8s4mm	0.77	Intercept			128.3	9.87
		Sphere A	−0.71	0.083	−0.26	0.03
		1−1/TB	−0.46		−62.02	11.31
4i8s6mm	0.91	Intercept			130.2	4.70
		Sphere A	−0.73	0.053	−0.26	0.02
		1−1/TB	−0.53		−57.1	5.66
4i8s8mm	0.87	Intercept			126.73	4.51
		Sphere A	−0.72	0.066	−0.25	0.02
		1−1/TB	−0.58		−46.1	5.29
8i8s4mm	0.74	Intercept			137.1	13.3
		Sphere A	−0.72	0.090	−0.27	0.03
		1−1/TB	−0.42		−70.54	15.14
8i8s6mm	0.87	Intercept			128.84	7.28
		Sphere A	−0.77	0.065	−0.28	0.02
		1−1/TB	−0.39		−53.8	8.88
8i8s8mm	0.87	Intercept			126.73	4.50
		Sphere A	−0.72	0.066	−0.25	0.02
		1−1/TB	−0.58		−46.1	5.29

**Figure 3:**
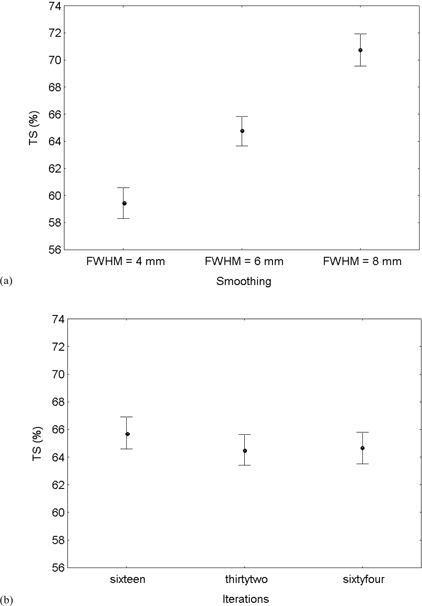
Sphere A ≤ 133 mm ^2^. TS averaged over all other variables for the different reconstruction strategies with increasing smoothing (a) or increasing iterations (b). Vertical bars indicate 95% confidence intervals of TS mean.

The results of the analysis of covariance are displayed in Table 3. TS means for the different reconstruction strategies were plotted together with 95% confidence intervals in Fig. 3. One point of the plot corresponds to the TS averaged over all other variables for one particular reconstruction scheme. TS averaged over target cross section A and (1−1/TB) are significantly different among the different amount of smoothing applied (p<0.01). The basis of this smoothing dependence can be appreciated from Fig. 3(a), where a higher threshold contour level must be used for smoother reconstructions in order to arrive at the same target volume. On the contrary, TS means are not significantly different among the different EM‐equivalent iterations used during OSEM reconstruction (p=0.20) (Fig. 3(b). Thus, we cannot reject the null hypothesis that different conditions of convergence during OSEM reconstruction do not influence threshold determination. In the plot, the nonvariable parameter values span the entire range of their variability. This accounts for the relatively wide range of mean TS values for each reconstruction scheme. Since the standard errors of the TS means were calculated on the basis of the common error term of the ANOVA table and since there are an equal number of observations in each class, the 95% confidence intervals are of the same magnitude for each point of the plot.

**Table 3:** Analysis of covariance results for sphere A $\leq \;133\;{\text{mm}}^{2}$.

*Effect*	*Sum of Squares*	*Degrees of Freedom*	*Mean Square*	*F*	*p*
Intercept	132283.2	1	132283.2	4199.8	<0.01
Sphere A	29231.1	1	29231.1	928.0	<0.01
(1−1/TB)	17106.0	1	17106.0	543.1	<0.01
Smoothing	5630.3	2	2815.1	89.4	<0.01
Iterations	84.3	2	42.1	1.33	0.26
Smoothing ^∗^Iterations	23.9	4	6.0	0.19	0.94
Error	8724.8	277	31.5		

The regression equation that best summarizes the results obtained pooling all the data corresponding to different OSEM iterations in a multiple regression model with TS as predicted variable and TB ratio, sphere A and amount of post‐reconstruction Gaussian smoothing as predictor variables may be written as:

(3) $$TS(\%)=55.94-0.25\times sphereA\;(mm^{2})+56.96\times \frac{1}{TB}+2.82\times FWHM\;(mm)$$

The adjusted coefficient of determination is $R^{2}=0.88$. The variables inserted into the model were statistically significant predictors of TS, whose variance can be accounted for, in order of decreasing relevance, by target dimensions $\left(\beta_{A}=-0.64\right)$, contrast $\left(\beta_{\left(1-1/\text{TB}\right)}=-0.52\right)$, and smoothing ($\beta_{\text{FWHM}}=0.29$).

### B. Algorithm for cross section A $>\;133\;{\text{mm}}^{2}$

The regression equation that best summarizes the results obtained in a multiple regression model with TS as predicted variable and TB ratio and sphere A as predictor variables may be written as:

(4) $$TS(\%)=B_{0}+B_{1}\times (1-\frac{1}{TB})$$

The adjusted coefficient of determination $R^{2}$, together with the estimated regression coefficients, were reported in Table 4 for each reconstruction strategy. Only (1−1/TB) resulted as statistically significant predictors of TS, while sphere A is no longer retained into the model as a significant predictor for each reconstruction algorithm examined. Again TS diminishes with increasing TB ratios. The coefficients of determination exhibit a tendency toward diminishing values with increasing EM‐equivalent iteration number. On the contrary, the increase of Gaussian smoothing seems to increase the goodness of fit of the selected reconstruction algorithm. The shrinkage on cross‐validation coefficient was calculated for the 2i8s8mm reconstruction scheme and amounted to 0.07.

**Table 4:** Results of multiple linear regression analysis with models fitted for sphere A $>\;133\;{\text{mm}}^{2}$.

*Reconstruction Strategy*	*R2*		β	*Standard Error of* β	*B*	*Standard Error of B*
2i8s4mm	0.91	Intercept			106.8	3.48
		1−1/TB	−0.95	0.051	−73.5	3.92
2i8s6mm	0.95	Intercept			105.1	2.30
		1−1/TB	−0.97	0.036	−70.1	2.60
2i8s8mm	0.94	Intercept			104.8	2.35
		1−1/TB	−0.97	0.038	−68.2	2.67
4i8s4mm	0.74	Intercept			83.5	6.69
		1−1/TB	−0.74	0.113	−47.3	7.23
4i8s6mm	0.88	Intercept			91.1	2.75
		1−1/TB	−0.94	0.054	−53.4	3.07
4i8s8mm	0.92	Intercept			97.5	2.41
		1−1/TB	−0.96	0.044	−59.5	2.72
8i8s4mm	0.90	Intercept			96.47	2.64
		1−1/TB	−0.95	0.048	−58.07	2.97
8i8s6mm	0.82	Intercept			85.27	3.11
		1−1/TB	−0.91	0.067	−46.7	3.48
8i8s8mm	0.90	Intercept			96.48	2.64
		1−1/TB	−0.95	0.049	−58.1	2.97

**Figure 4:**
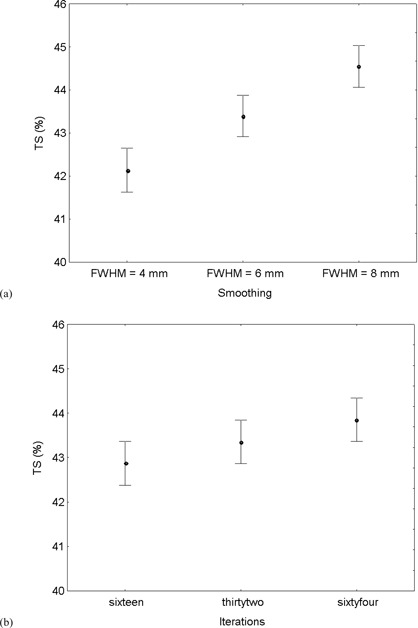
Sphere A > 133 mm ^2^. TS averaged over all other variables for the different reconstruction strategies with increasing smoothing (a) or increasing iterations (b). Vertical bars indicate 95% confidence intervals of TS means.

The results of the analysis of covariance are displayed in Table 5. TS means for the different reconstruction strategies were plotted together with 95% confidence intervals in Fig. 4. Also in this case TS averaged over target cross section A and (1−1/TB) are significantly different among the different amount of smoothing applied (p<0.01), with more smoothing requiring a higher percentage threshold. (Fig. 4(a). Again, TS are not significantly different among the different EM‐equivalent iterations used during OSEM reconstruction (p=0.02), although a tendency toward an increase in TS with increasing convergence of the OSEM algorithm can be appreciated form Fig. 4(b). No significant interaction was observed between smoothing and iterations (p=0.76).

**Table 5:** Analysis of covariance results for sphere A $>\;133\;{\text{mm}}^{2}$.

*Effect*	*Sum of Squares*	*Degrees of Freedom*	*Mean Square*	*F*	*p*
Intercept	51625.7	1	51625.7	7009.3	<0.01
Sphere A	17.5	1	17.5	2.4	0.12
(1−1/TB)	16680.1	1	16680.1	2264.7	<0.01
Smoothing	330.5	2	165.3	22.4	<0.01
Iterations	56.8	2	28.4	3.9	0.02
Smoothing ^∗^Iterations	13.6	4	3.4	0.5	0.76
Error	2541.0	345	7.4		

The regression equation that best summarizes the results obtained pooling all the data corresponding to different OSEM iterations in a multiple regression model with TS as predicted variable and TB ratio and amount of post‐reconstruction Gaussian smoothing as predictor variables may be written as:

(5) $$TS(\%)=33.13+60.27\times \frac{1}{TB}+0.60\times FWHM\;(mm)$$

The adjusted coefficient of determination is $R^{2}=0.87$. The variables inserted into the model were statistically significant predictors of TS, whose variance can be accounted for, in order of decreasing relevance, by contrast $\left(\beta_{\left(1-1/\text{TB}\right)}=-0.91\right)$, and smoothing $\left(\beta_{\text{FWHM}}=0.13\right)$.

### C. Technical delineability

**Figure 5:**
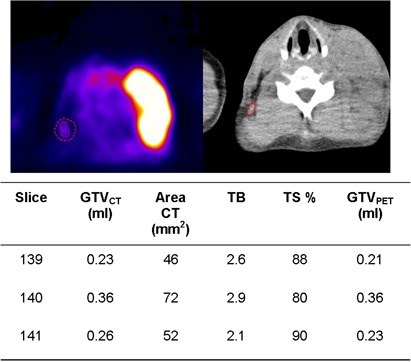
Example showing a good agreement between ${\text{GTV}}_{\text{PET}}$ (0.8 ml) and ${\text{GTV}}_{\text{CT}}$ (0.9 ml) in a small lymph node with faint uptake, immersed in a uniform background. Also shown is derivation of the ${\text{GTV}}_{\text{PET}}$.

The case represented in Fig. 5 is a spinal lymph node with FDG accumulation in a patient with Hodgkin's lymphoma. Its axial extension involves only three 2 mm thick slices. In these axial slabs both the cross‐sectional areas of the lymph node and the TB ratio are relatively small and uniform, ranging from 46 to 72 mm^2^ and from 2.1 to 2.9, respectively. In all of the slices, Eq. (3) has been applied. As a consequence, the TS for auto‐contouring these slices are elevated, showing only a slight variation comprised between 80% and 90%. When analyzing volume sizes, only a small difference is detected between ${\text{GTV}}_{\text{PET}}$ (0.8 ml) and ${\text{GTV}}_{\text{CT}}$ (0.9 ml), which amounts to a −11% relative difference.

**Figure 6:**
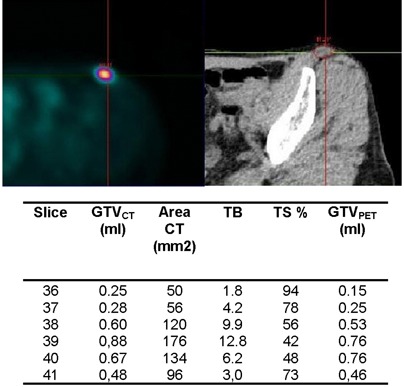
Example showing a good agreement between ${\text{GTV}}_{\text{PET}}$ (2.9 ml) and ${\text{GTV}}_{\text{CT}}$ (3.2 ml) in a medium sized lymph node with variable cross‐sectional dimensions, showing a highly variable TB ratio in different slices. Also shown is derivation of the ${\text{GTV}}_{\text{PET}}$.

The case represented in Fig. 6 is an inguinal lymph node with FDG accumulation derived from a primitive melanoma. Its axial extension involves six 2 mm thick slices. In these axial slabs, the cross section areas exhibit a significant variation comprised between 50 and 176 mm^2^. In the first three and the last slices, the relationship in Eq. (3) has been applied, while in the fourth and fifth slices, the relationship in Eq. (5) holds. In this case, a significant variation in the TB ratio is observed across different slices from 1.8 to 12.8. As a consequence, the TS for auto‐contouring these slices span almost the entire range of variability, ranging from 42% and 94%. When analyzing volume sizes again, a small difference is detected between ${\text{GTV}}_{\text{PET}}$ (2.91 ml) and ${\text{GTV}}_{\text{CT}}$ (3.16 ml), which amounts to a −8% relative difference.

In Table 6 the individual comparisons of bladder volumes determined in five patients on CT and using the adaptive threshold algorithm on PET, images are reported. A mean relative difference of −5.1% (range −13.4% to +3.7%) was found between CT and PET volumes.

**Table 6:** Comparisons of bladder volumes determined in five patients on CT and using the adaptive threshold algorithm on PET images.

*Patient*	*CT Volume (mL)*	*TB Ratio (min‐max)*	*TS (%) (min‐max)*	*PET Volume (mL)*	*Deviation from CT (%)*
1	313.1	3–14	42–59	313.1	0
2	130.3	3–18	40–57	112.9	−13.4
3	171.9	4–29	39–54	156.0	−9.3
4	41.9	8–12	42–45	43.4	+3.7
5	287.9	3–16	41–57	268.6	−6.7

## IV. DISCUSSION

Various basic approaches were reported in literature to accurately contour FDG‐based gross target volumes for subsequent use in radiation treatment planning. Among them, the most promising in terms of ease of application (possibility to be implemented in software installed on treatment planning systems and validation in phantom and patients) are contrast‐oriented algorithms which usually assume a linear relationship between TS and 1/TB ratio.^(^ ^2^ ^–^ ^4^ ^)^ On the other hand, it is now widely recognized that TS depends also on target size, but this dependence is only apparent when the full range of target size is considered.^(^ ^8^ ^–^ ^10^ ^)^ This size dependence is particularly pronounced for smaller targets due to partial volume effect, which is well documented in the imaging literature.^(22)^ Thus, it seems reasonable to assume that two different functional forms need to be fitted for accurate TS determination: one for small volumes and the other for volumes exceeding a value which is somewhat dependent on the resolution characteristics of the scanner used. Partial volume effects significantly reduce the contrast recovery for structures less than two or three times the reconstructed spatial resolution^(23)^ which in our scanner is about 4.5 mm. Thus the choice of sphere $A=133\;{\text{mm}}^{2}$ (or, equivalently, a sphere internal diameter of 13 mm) as a separator of the data was dictated by the resolution characteristics of our scanner. Another reason for splitting the functional form of the adaptive thresholding algorithm is that there might have been a difference in the convergence rate of the recovered activity concentration as a function of target size.

The central idea behind the use of adaptive thresholding is its optimization based on contrast, target size and overall clinical image quality for each scanner. The reconstruction parameters for diagnosis are optimized in terms of image quality, considering the relative noise/smoothing in association with overall acquisition duration and system sensitivity, quantitative accuracy, etc. However, the use of FDG‐PET images for radiotherapy planning requires that identical patient position is ensured during planning CT and PET scan. This task is accomplished through the use of the same positioning aids on both modalities. This in turn requires that a separate PET acquisition must be performed in radiotherapy treatment position. It is thus likely that there will be two reconstruction settings used in a single center: one for clinical use and reporting, and another whose optimization can be based on the performance of a segmentation algorithm.

It has been previously demonstrated that more smoothing during reconstruction requires a higher threshold contour level.^(^ ^2^ ^,^ ^8^ ^)^ Among the imaging parameters that may have a significant impact on TS determination and have not yet been investigated there are the number of OSEM iterations.

**Figure 7:**
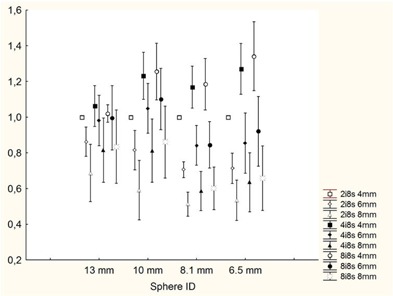
Maximum values for the different reconstruction schemes in the sphere ID=10 mm. The values of the maxima have been normalized to the maximum with 2i8s4mm reconstruction scheme.

The results of our study show that the degree of convergence of OSEM algorithms does not influence TS determination for target cross‐sectional areas lower than 133 mm^2^, while only a slight tendency toward an increase of TS with increasing iterations can be observed for bigger targets. However, this is always a second‐order effect in comparison to smoothing. This finding is not intuitive: one would expect that increasing the number of iterations would increase the maximum pixel in a ROI and, hence, would affect the threshold settings. The results of this paper however show otherwise. One reason could be that the extent of smoothing used impacted the PET images more than the extent of equivalent iterations. Figure 7 shows the maximum values for the different conditions normalized to the maximum obtained at 2i8s4mm for sphere IDs in the range of 6.5–13 mm, where partial volume effects are relevant and both equivalent iterations and the amount of smoothing are expected to affect the maximum value in the target. One can easily check that the decrease of the maximum value with increasing smoothing is much faster than its increase with increasing equivalent iterations.

The inclusion of smoothing in the adaptive thresholding algorithm adds to the generalization of the method allowing its use in centers equipped with the same scanner but using a different set of reconstruction parameters.

Bearing this in mind, we can now answer another interesting question: Which is the set of reconstruction parameters that should be chosen for this specific scanner in order to maximize the accuracy of fitting? The coefficient of determination $R^{2}$ is a well‐known metric in statistics that provides a measure of how well future outcomes are likely to be predicted by the model. Looking at Tables 2 and 4, the choice is restricted between the 2i8s6mm and 2i8s8mm reconstruction algorithms, both providing a very high $R^{2}=0.90$ for A ≤ 133 mm ^2^ and $R^{2}=0.95$ for A > 133 mm ^2^. The choice between them is thus driven by a different requisite of algorithms aimed at prediction: robustness.

A largely debated question in the process of devising algorithms for PET‐based GTV delineation is how to define the threshold contour level. Black et al.^(24)^ suggested that the threshold contour level should be set at a fraction of the mean uptake of the lesion. As pointed out by Ford et al.,^(8)^ this is necessarily an iterative process since a region of interest must be first defined in order to calculate the mean uptake, and the mean uptake depends on the size of the region of interest. Schaefer et al.^(6)^ used a threshold of 70% of the maximum standardized uptake value of the lesion, to take into account the inhomogeneity of the FDG accumulation. Jentzen et al.^(4)^ used the maximum activity concentration but, to remove statistical outliers, the values from adjacent slices were considered using a parabolic fit around the maximum. For small spheres, the Gaussian amplitude was used as the source activity concentration. Daisne et al.^(2)^ defined the maximal activity as the average activity of nine voxels surrounding the hottest voxel. Davis et al.^(25)^ used maximum activity for sources of diameter ≤ 12.5 mm and the mean activity of the highest 10% adjacent pixels for signals with a diameter > 12.5 mm. In this and other previous studies,^(^ ^8^ ^–^ ^9^ ^)^ the threshold has been defined as a fraction of the maximum voxel value.

It must be recognized that the functional dependence of TS contour level on target size, TB ratio and reconstruction smoothing are expected to be roughly of equal magnitude whether one uses the maximum or some form of averaged uptake. On the other hand, it must also be recognized that, in order to minimize the influence of nonrepresentative global maximum values which are subjected to statistical variation, an increase in the amount of reconstruction smoothing has the same effect of taking some form of averaged uptake. What matters in the context of algorithms aimed at prediction is model goodness of fit (that is $R^{2}$) and robustness. In this respect, Gaussian smoothing seems preferable to the variety of alternative strategies suggested, at least for ease of implementation and need of standardization. Thus, the choice of 2i8s8mm reconstruction algorithm in our scanner has been dictated by the need of robustness.

Another point that deserves further discussion is the reliability of the selected regression model. Having chosen a model that is best for a particular sample of data, one has no assurance that such a model can be reliably applied to other samples. Most generally accepted methods for assessing model reliability involve some form of split‐sample approach, where the regression model is tested against a “validation” sample that was not used in model building. Typically $R^{2}{}_{*}$, the cross validation correlation, is a less‐biased estimator of the population squared multiple correlation than is the (positively) biased $R^{2}$. Hence, the shrinkage statistic is almost always positive. How large must shrinkage be to cast doubt on model reliability? No firm rules can be given, but shrinkage values of less than 0.10 are indicative of a reliable model, and this is indeed our case.

Another relevant point that deserves further consideration is the choice of a cross section for the analysis instead of a volume approach. As shown by Drever et al.^(9)^ no single threshold value will simultaneously yield both a correct determination of total target volume and also individual cross section for objects of variable cross‐sectional shape. Only a slice‐specific threshold level can come closest to correctly reproducing both correct cross section and the total volume of a structure. Although spherical objects were used in the present study to derive the coefficients of the algorithm (and in this configuration, a cross section and a volume approach are likely to come to the same conclusion), the question of how to properly define target volumes is crucial to the efficacious quantitative use of the functional information provided by PET in radiotherapy planning, and for future validation of the algorithm both in phantom and in patients. The practical utility of an optimal single threshold derived from a volumetric approach is questionable at best as it would apply only to spherical volumes. It must be underlined that both approaches provided a volume determination. The only difference is that in the slice‐specific approach, the volume is determined as the sum of different slabs in which segmentation is based upon the slice‐specific contrast between target and surrounding background.

It must be emphasized that the results of this study are generalized to different image acquisition protocols characterized by different statistical count levels in acquired sinograms, since neither emission scan duration nor background activity concentration play any role in adaptive threshold algorithm derivation.^(10)^ Moreover, the algorithms described in Eqs. (3) and (5) are not specific to the particular phantoms used in their derivation since adaptive threshold algorithms were demonstrated to be largely independent on the conditions of attenuation and scatter.^(11)^ The feasibility of the method was illustrated in two patients with FDG nodal accumulation in which the CT volume of the lymph nodes served as a gold standard, with satisfactory results. The applicability of such a localized targeting technique on large areas of tissue simulating large tumors or large areas of tissues to be included in a prophylactic CTV was illustrated in five patients in whom the CT volume of the bladder served as a gold standard, with satisfactory results.

### A. Study limitations

The results of this study must be interpreted in the context of several limitations.

Although the method is uniformly applicable, the values of parameters for Eqs. (3) and (5) reported in this study are system‐dependent. These values have to be separately adjusted for each scanner and for each reconstruction algorithm by phantom measurements, as described in this study.

The effects of lesion movement in lung tumors have been recently incorporated in an adaptive thresholding algorithm using multiple regression techniques similar to those in the present study.^(26)^ A different approach for incorporating respiration mobility into radiotherapy planning is tracking the tumor, for instance with 4D‐PET/CT,^(27)^ and delivering treatment at a particular phase of respiratory gating or dynamically so as to follow the tumor changing position.^(28)^ The effect of respiratory motion is a blurring of the tumor on the resulting image. Activity is spread out over voxels in the motion path of the tumor leading to decreased TB ratio and increased dimensions. Though the effects of lesion movement were not included in this study, we believe that the conclusions regarding the effect of smoothing and iterations on thresholds still apply in the case of moving targets due to the wide range of lesion sizes and TB ratios examined.

Threshold techniques do no take into account variations in tumor heterogeneity resulting in under‐ or overestimation of the tumor extent depending on the selected threshold values. This has motivated the investigation of gradient‐based segmentation techniques based on gradient differences between the foreground lesion and the background. These include simple edge or ridge detectors such as the Sobel operator and the Watershed transform evaluated by Drever et al.^(29)^ They reported that direct application of the Sobel edge detector or the Watershed transform failed to correctly identify the correct size of experimental volumes compared with thresholding methods. This is in contrast with the results of Geets et al.^(30)^ in which the Watershed transform was applied in conjunction with cluster analysis on heavily preprocessed images. While referring to these important methods for segmentation of nonuniform tracer concentration, it should be pointed out that until they are further developed and validated, adaptive threshold segmentation methods are and will be used in most clinics and therefore need to be accurately characterized.

## V. CONCLUSIONS

The results of our study show that the degree of convergence of OSEM algorithms does not influence TS determination. More smoothing during reconstruction requires a higher percentage threshold contour level. Among the reconstruction schemes investigated, the OSEM at two iterations and eight subsets with a 6–8 mm post‐reconstruction Gaussian three‐dimensional filter provided the better goodness of fit with a coefficient of determination $R^{2}=0.90$ for cross‐sectional areas ≤ 133 mm ^2^ and $R^{2}=0.95$ for cross‐sectional areas > 133 mm ^2^. OSEM at two iterations and eight subsets with an 8 mm postfiltering was selected as the best performing algorithm due to robustness considerations. The inclusion of post‐reconstruction smoothing in the adaptive thresholding algorithm adds to the generalization of the method.

## ACKNOWLEDGMENTS

This work was supported by a grant from the Piedmont Region, Italy in the frame of “Ricerca Sanitaria Finalizzata 2008”.
